# Pi*SCP1* and Pi*CDPK2* Localize to Peroxisomes and Are Involved in Pollen Tube Growth in *Petunia Inflata*

**DOI:** 10.3390/plants2010072

**Published:** 2013-03-04

**Authors:** Feng Guo, Gyeong Mee Yoon, Andrew G. McCubbin

**Affiliations:** 1 Department of Molecular, Cellular, and Developmental Biology, University of Michigan, Ann Arbor, MI 48109, USA; E-Mail: fengguo@umich.edu; 2 Department of Biology University of North Carolina, Chapel Hill, NC 27599, USA; E-Mail: gyoon@email.unc.edu; 3 School of Biological Sciences and Center for Reproductive Biology, Washington State University, Pullman, WA 99164, USA

**Keywords:** pollen tube growth, calcium dependent protein kinase, peroxisome

## Abstract

*Petunia inflata* small CDPK-interacting protein 1 (PiSCP1) was identified as a pollen expressed PiCDPK1 interacting protein using the yeast two hybrid system and the interaction confirmed using pull-down and phosphorylation assays. PiSCP1 is pollen specific and shares amino acid homology with uncharacterized proteins from diverse species of higher plants, but no protein of known function. Expression of PiSCP1-GFP *in vivo* inhibited pollen tube growth and was shown to localize to peroxisomes in growing pollen tubes. As PiCDPK1 is plasma membrane localized, we investigated the localization of a second isoform, PiCDPK2, and show that it co-localizes to peroxisomes with PiSCP1 and that the two proteins interact in the yeast 2 hybrid interaction assay, suggesting that interaction with the latter CDPK isoform is likely the one of biological relevance. Both PiCDPK2 and PiSCP1 affect pollen tube growth, presumably by mediating peroxisome function, however how they do so is currently not clear.

## 1. Introduction

Ca^2+^ has long been known to play a key regulatory role in pollen tube growth [1,2,3,4]. A steep tip-focused free Ca^2+^ gradient is found in growing tubes, and its experimental dissipation leads to a reversible cessation of growth [2,5,6]. Recently, considerable progress has been made in identifying the channels by which Ca^2+^ enters the pollen tube cytosol (for reviews see [7,8]). There is a wealth of evidence to show that calcium entry and the free Ca^2+^ gradient are regulated by a class of plant specific Rho GTPases, designated Rop/Rac’s (Rho-like GTPase of plants) [9,10,11,12]. A considerable array of signaling molecules have been implicated in the regulation of the highly polar, tip focused growth in pollen tubes of which Rop/Rac’s are regarded as central regulators (for recent review see [13]), regulating not only the tip-focused Ca^2+^ gradient but also the apical actin cytoskeleton through antagonistic effector pathways [14].

One class of potential downstream effectors of Ca^2+^ signaling in pollen tubes is calcium dependent protein kinases (CDPKs). Depolarized growth has been reported to result from over-expression of a petunia CDPK isoform (*PiCDPK1*) in pollen, whereas a constitutively active mutant version of this isoform severely inhibited growth [15]. Further, double T-DNA insertion mutants of Arabidopsis CPK-17 and -34 (the CDPK isoforms most closely related to *PiCDPK1* in the Arabidopsis), exhibited a ~3 fold reduction in growth rate and 350 fold reduction in transmission efficiency [16]. Though the immediate downstream target of these CDPK’s is unknown, over-expression of *PiCDPK1* caused a dramatic increase in tip focused Ca^2+^ concentration, suggesting the involvement of a positive feedback loop [15]. The CDPK isoforms mentioned above appear to be involved in regulating the polarity of pollen tube growth, there are however, additional isoforms expressed in pollen tubes. Experiments using Arabidopsis gene chips have identified 16 of the 34 CDPK isoforms in the Arabidopsis genome as being expressed in pollen, six at high levels (*AtCPKs* *14*, *16*, *17*, *24* and *34*) [17]. Over-expression of Petunia *PiCDPK2* (a homolog of *AtCPK24*) has been shown to inhibit pollen tube extension [15] but not affect growth polarity, and in addition localized to an underdetermined internal membrane compartment as opposed to PiCDPK1, which localized to the plasma membrane [15]. Though most commonly plasma membrane associated, CDPKs have been shown to localize to a variety of intracellular location including the cytosol, nucleus, endoplasmic reticulum, peroxisomes, mitochondrial outer membrane and oil bodies [16,17,18,19,20,21,22]. Either by direct demonstration, or inference based on functional commonality with PiCDPK1, *AtCPKs* *16*, *17* and *34* localize to the plasma membrane [15,22], but the cellular locations of *AtCPK14* and *24* (and the *AtCPK24* homolog PiCDPK2) remain to be determined, as do their roles in pollen tube growth.

The downstream targets of CDPK activities involved in pollen tube growth are not known, and identification of CDPK substrates is an important goal in elucidating the signaling pathways in which they are involved. As plants appear to employ a combination of strategies to functionally specialize individual CDPKs, including tissue-specific distribution, subcellular localization, and enzyme kinetics and properties [23,24] it is important to recognize that methods for identifying substrates are based on biochemical interaction. With this in mind we sought to identify CDPK substrates in pollen tubes by screening a pollen cDNA yeast 2 hybrid library using the kinase domain of PiCDPK1 as bait and report the identification and characterization of Petunia inflata *Small CDPK interacting Protein 1* (Pi*SCP1*), and demonstrate that its protein product co-localizes to peroxisomes with PiCDPK2.

## 2. Results and Discussion

### 2.1. Identification of a cDNA Clone Product That Interacts with the PiCDPK1 Kinase Domain

The GAL4 yeast two hybrid system was used to identify proteins that interact with PiCDPK1. The kinase domain of PiCDPK1 with a 6 amino acid *N*-terminal deletion was used a bait construct to avoid possible problems resulting from putative myristoylation and palmitoylation sites at the *N*-terminus [15]. The bait construct was tested for ability to auto-activate the histidine (HIS) nutritional reporter and found not to activate the reporter. Approximately 2 × 10^7^ yeast cells were transformed with the kinase bait construct and a *Petunia inflata* pollen cDNA library in the yeast 2-hybrid prey vector. After several rounds of selection with appropriate controls, 4 classes of interacting clones were identified and sequenced. Only one class was found to encode an insert that created an in-frame fusion, this was transformed back into yeast, and re-assayed for activation of ß-gal, alone and in combination with the bait construct. These controls confirmed that the interaction was reproducible and not a result of auto-activation or genomic mutation. The clone obtained was a partial cDNA and was used to screen a pollen cDNA library to identify a full-length cDNA that we named *P. inflata* Small CDPK interacting Protein 1 (Pi*SCP1*). Pi*SCP1* encodes a protein of 103 amino acids with a deduced molecular mass of 11.7 kDa (GenBank accession# KC342807).

**Figure 1:**
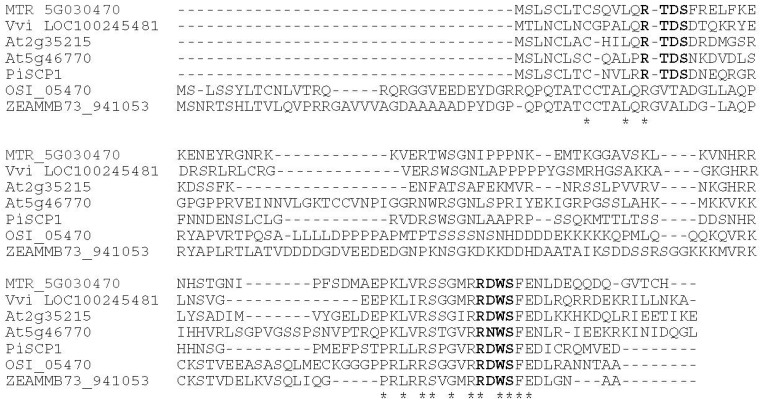
Comparison of amino acid sequences of PiSCP1 homologs. Sequences were aligned using ClustalW2. Amino acids conserved among all sequences are underscored with an asterisk. Conserved potential CDPK phosphorylation sites are indicated in bold. MTR, *Medicago trunculta*; Vvi, *Vitis vinifera*; At, *Arabidopsis thaliana*; OSI, *Oryza* *sativa*; ZEAMMB, *Zea mays*.

BLAST searches with Pi*SCP1* revealed significant homology to a number of uncharacterized genes form higher plants, including 2 *Arabidopsis* expressed genes, but no gene of known function. Alignment with a selection of these genes revealed two conserved regions separated by a highly variable central region. The first, at the *N*-terminus being conserved in eudicot species and the second at the *C*-terminus being conserved in both eudicot and monocot species (Figure 1). The PiSCP1 protein contains multiple potential sites of phosphorylation by CDPK, based on the consensus Basic-X-X-S/T [25], the *N*- and *C*-terminal conserved regions each possess one site of potential serine phosphorylation which is conserved between the homologs (Figure 1). One of the *Arabidopsis* homologs (At5g46770) has been classified as pollen-enriched by microarray analysis [26].

### 2.2. PiCDPK1 Interacts with and Phosphorylates PiSCP1 *in Vitro*

**Figure 2:**
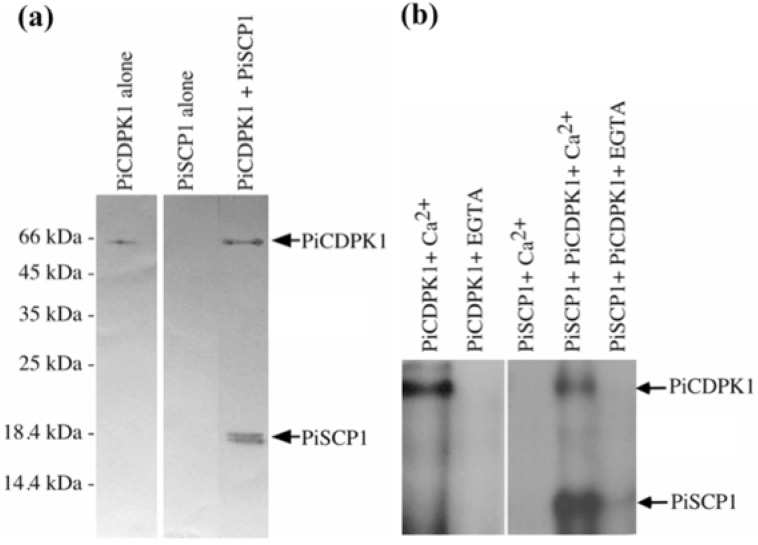
PiCDPK1 interacts with and phosphorylates PiSCP1. (**a**) *In vitro* binding assay with PiCDPK1. His-tag-PiSCP1 fusion protein was incubated with phenyl-sepharose bound His-tag-PiCDPK1 protein in the presence of 50 µM Ca^2+^. After washing, the bound fraction was separated by SDS-PAGE and subjected to Western blotting using anti-His antibody to detect His-tag fusion proteins. (**b**) *In vitro* phosphorylation assay of PiSCP1 by PiCDPK1 in the presence of 50 µM Ca^2+^ or 1 mM EGTA.

To confirm the interaction between PiCDPK1 and PiSCP1 proteins, we performed an *in vitro* binding assay with purified fusion proteins of PiCDPK1 and PiSCP1. Each protein was expressed as a 6× His tagged fusion protein in *E. coli* and purified using Ni-NTA^+^ resin. PiSCP1 was then incubated with phenyl sepharose resin-bound His tagged PiCDPK1 or phenyl sepharose resin alone, in the presence of Ca^2+^ or EGTA. Phenyl sepharose resin binds specifically to proteins containing a calmodulin-binding domain in the presence of Ca^2+^ [27], facilitating pull down of PiCDPK1 but not PiSCP1 except through interaction with PiCDPK1. After washing, the resin-bound fraction was washed, eluted and subjected to Western blot using an anti-His tag monoclonal antibody. As shown in Figure 2a, PiSCP1 was pulled down only in the presence of PiCDPK1, demonstrating that it did indeed interact with PiCDPK1 in this assay was well as in the yeast 2 hybrid system. 

We next assayed whether PiCDPK1 was able to phosphorylate PiSCP1 *in vitro*. As shown in Figure 2b, PiSCP1 was not phosphorylated in control samples lacking PiCDPK1 or in the presence of PiCDPK1 without Ca^2+^, but was phosphorylated *in vitro* in the presence of PiCDPK1 and Ca^2+^, consistent with PiSCP1 being a substrate of this protein kinase.

### 2.3. Expression Profile of PiSCP1

**Figure 3:**
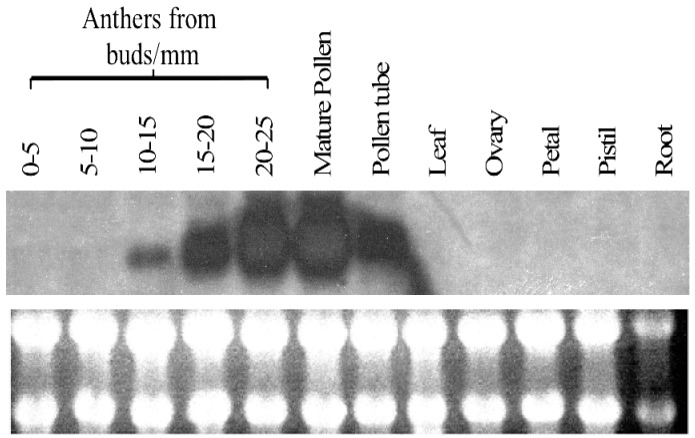
Expression profile of *PiSCP1.*

*PiSCP1* was identified from a mature pollen cDNA library. To assess the tissue specificity of the expression of this gene as well as determine developmental regulation in pollen development *PiSCP1* gene expression was examined by RNA gel blot analysis (Figure 3). The transcript was found to be pollen-specific, expression starting late in pollen development (10 mm buds = pollen mitosis I), peaking in mature pollen and remaining high in germinated pollen tubes. This expression pattern is almost identical to those of *PiCDPK1* and *PiCDPK2* [15], consistent with the products of these genes being present at the same time in pollen development as well as in pollen tubes.

### 2.4. Transient Over-Expression of PiSCP1 in Pollen Tubes

**Figure 4:**
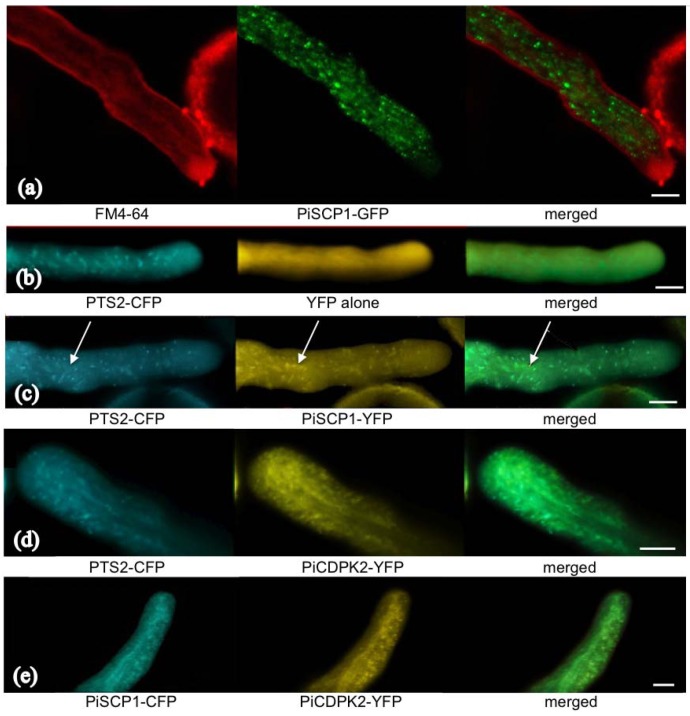
Localization of PiSCP1 and PiCDPK2. Panels indicate fluorescence imaging of FM4-64 and fluorescent protein constructs, the identity of which is indicated below each image. Merged panels were produced by combining the two images immediately to the left of each. All images were generated using a confocal microscope. Scale bars = 5 µm.

We transiently expressed PiSCP1-GFP in pollen tubes to investigate whether it would generate a phenotype and investigate sub-cellular localization. The fusion protein was found to cause significant inhibition of pollen tube growth, transformed tubes only growing to 56.5% (n = 50, s.d. 5.6%) of the length of untransformed tubes (from the same samples). Florescence associated with the fusion protein was found to localize to internal membrane compartments, mostly small spherical organelles and some slightly larger organelles (Figure 4a), which moved rapidly with cytoplasmic streaming. Counter staining with FM 4-64 showed that these small organelles largely stay out of the apical clear zone and are not stained by FM 4-64 (Figure 4a), indicating that these organelles are most likely not part of endocytic pathways.

The localization observed for PiSCP1 was reminiscent of previously reported peroxisome localization in pollen [22]. To test whether PiSCP1 localized to peroxisomes, a co-localization experiment was performed using PiSCP1-YFP and cyan fluorescent protein (CFP)-tagged with the peroxisome-targeting signal PTS2 of an *Arabidopsis* thiolase [28]. Thiolase is a classical PTS2 peroxisomal protein first identified in yeast [29]. Co-expression of this peroxisome marker with a vector encoding soluble YFP alone confirmed that in our hands the localization of this marker was identical to published results and that we were able to image CFP and YFP fluorescence separately without “bleed-over” fluorescence (Figure 4b). When PTS2-CFP and PiSCP1-YFP were co-expressed, extensive co-localization was observed (Figure 4c), though some larger compartments emitted a PiSCP1-YFP signal but not PTS2-CFP fluorescence (indicated by arrows), the identity of these compartments is currently not known. 

### 2.5. PiCDPK2 Localized to Peroxisomes and Co-Localized with PiSCP1

Though PiSCP1 was identified as a protein that interacted with PiCDPK1, we have previously shown that PiCDPK1 exhibits plasma membrane localization that is dependent on potential myristoylation and palmitoylation sites at the *N*-terminus, however a second isoform—PiCDPK2, exhibits localization pattern reminiscent of that of PiSCP1 [15]. As the identity of the membrane compartment that PiCDPK2 associated with had not been determined, we performed further co-localization experiments between PiCDPK2-YFP and either the peroxisome marker or PiSCP1-CFP. Interestingly, PiCDPK2-YFP did co-localize with both PTS2-CFP and PiSCP1-CFP and hence localized to peroxisomes, however the peroxisomes appeared to be somewhat modified by its presence, as the fluorescent signal generated by the targeted fluorescent proteins (both the PTS2-CFP marker and PiSCP1-CFP) was consistently more diffuse when PiCDPK2 was present, causing visible co-localization to be less distinct (Figure 4d,e). In addition in the presence of PiCDPK2, peroxisomes were observed to enter the tip region, from which they are usually excluded (Figure 4d,e) [30].

### 2.6. PiSCP1 Interacts with PiCDPK2 in the Yeast 2 Hybrid System

**Figure 5:**
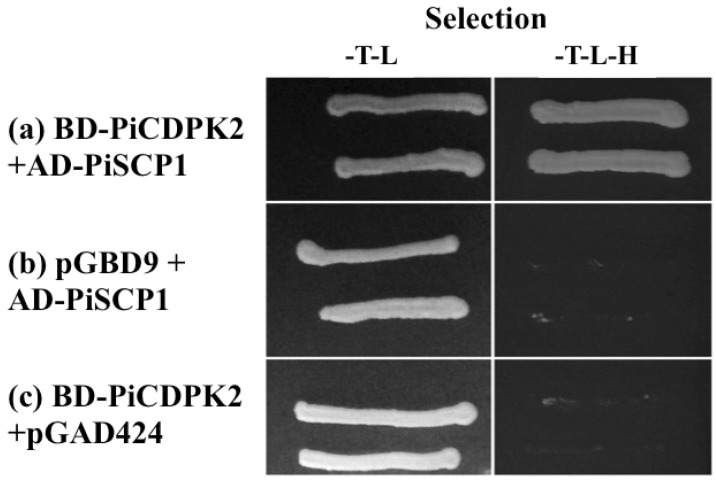
PiSCP1 interacts with PiCDPK2 in yeast. Yeast transformed with bait and prey constructs, was grown on selection media, either SD/-T-L (plasmid selection) or SD/-T-L-H (interaction selection). (**a**) Yeast containing BD-PiCDPK2 and AD-PiSCP1 grow on both plasmid and interaction selecting media. (**b**) and (**c**) Neither BD-PiCDPK2 nor AD-PiSCP1 with their respective empty partner vectors was able to auto-active Histidine reporter gene.

As localization appears to be at least as, if not more, important than primary structure in determining the substrate specificity of CDPKs [25] co-localization of PiSCP1 with PiCDPK2 suggests that despite being identified by *in vitro* interaction with PiCDPK1, PiCDPK2 is more likely to be isoform interacting with PiSCP1 *in vivo*. Unfortunately, despite extensive efforts we were unable to express active PiCDPK2 in *E. coli*, hence to investigate putative interaction between PiSCP1 and PiCDPK2 we employed the yeast two hybrid system. We determined that PiSCP1 did interact with the *N*-terminal variable/kinase domain of PiCDPK2 to facilitate yeast growth under His selection, whereas neither PiSCP1 nor the *N*-terminal variable/kinase domain of PiCDPK2 alone was able to do so (Figure 5). Hence PiSCP1 interacted with the *N*-terminal variable/kinase domains of both pollen expressed CDPK isoforms despite these regions exhibiting only 21% amino acid identity overall and this identity being scattered through the domains [31]. As PiSCP1 and PiCDPK2 both localized to peroxisomes we suggest that most likely this is the interaction of physiological relevance. That our results suggest both PiCDPK1 and PiCDPK2 interact with PiSCP1 is significant. The kinase domains of CDPKs are highly conserved leading to speculation that isoform specificity may be encoded by tissue-specificity, subcellular localization, or enzyme kinetics/properties rather than primary structure [23,24]. However, recent results have shown that differences in substrate specificity between some CDPK isoforms is encoded by the sequence of the *N*-terminal variable domain [32]. The results presented here support the hypothesis that, at least for some isoforms, localization may be the most significant factor in determining specificity *in vivo*. It is interesting to note that the isoform for which the *N*-terminal variable domain determines specificity is plasma membrane localized [32], as are the majority of CDPK isoforms investigated to date [22]. As many isoforms exhibit this localization, this property alone is obviously insufficient to provide substrate specificity when comparing any individual pair. Conversely, to date only one isoform has been shown to exhibit peroxisomal localization in a particular cell type [22], hence the need for an additional mechanism(s) for generating substrate specificity between these and other isoforms is greatly reduced or absent. Thus we speculate that the mechanism generating substrate specificity between isoforms *in vivo* may in fact depend on the characteristics of the isoforms in question, in particular whether they are co-expressed in the same cell type(s) and exhibit the same cellular localization.

Peroxisomes have recently emerged as organelles of critical importance in sexual plant reproduction. Functional peroxisomes on either the male or female side of the pollen-pistil interactions are a prerequisite for fertilization [33]. These organelles possess rich enzymatic machinery and have been implicated in many developmental processes in Arabidopsis [34,35,36]. In pollen, peroxisomes are the main site of intracellular nitrous oxide (NO) production [31]. Extracellular NO has been reported to be important in targeting pollen tube growth to ovules [37] as well as in pollen stigma interactions [38] and NO has been shown to stimulate pollen tube growth orientation [30]. Within pollen tubes, peroxisomes are excluded from the pollen tube tip and low NO within this region is a pre-requisite for growth [30]. Both cGMP and Ca^2+^ have been implicated in NO regulated pathways involved in the growth re-orientation. Stimulation of pollen tube tips with NO leads to two peaks of cytosolic Ca^2+^, one associated with an initial cessation of growth and a second with re-orientation and regrowth [37]. Given this link between peroxisomes, NO and Ca^2+^, it is tempting to hypothesize that PiCDPK2, being Ca^2+^ regulated and peroxisome localized, might be a component of a feedback loop. That PiCDPK2 over-expression both led to inhibition of tube growth and caused peroxisomes to enter the tip region (Figure 4c,d) is consistent with this scenario [15], however if such a feedback loop existed (either positive or negative) over-expression of PiCDPK2 would be predicted to alter the intracellular tip-focused Ca^2+^ gradient. We have previously shown that over-expression of PiCDPK2 (in contrast to the plasma membrane localized isoform PiCDPK1) had no affect on this gradient [15], leading us to conclude that PiCDPK2 is unlikely to participate in a feed back loop with NO and Ca^2+^.

Information on Ca^2+^ signaling in peroxisomes is scarce and was only recently recognized in animal cells [39]. In plants, Ca^2+^ has been shown to stimulate detoxification of the reactive oxygen species H_2_O_2_ by calmodulin activation of AtCAT3 activity within peroxisomes [40], and peroxisome localized AtCPK1 has been shown to mediate pathogen resistance [41]. The lack of homology of PiSCP1 to any protein or domain of known function prevented us from hypothesizing its function in pollen tubes and in an effort to address this problem we performed an additional yeast two hybrid screen using PiSCP1 as bait in an attempt to identify proteins acting downstream of PiSCP1.

### 2.7. PiSCP1 Forms Multimers

**Figure 6:**
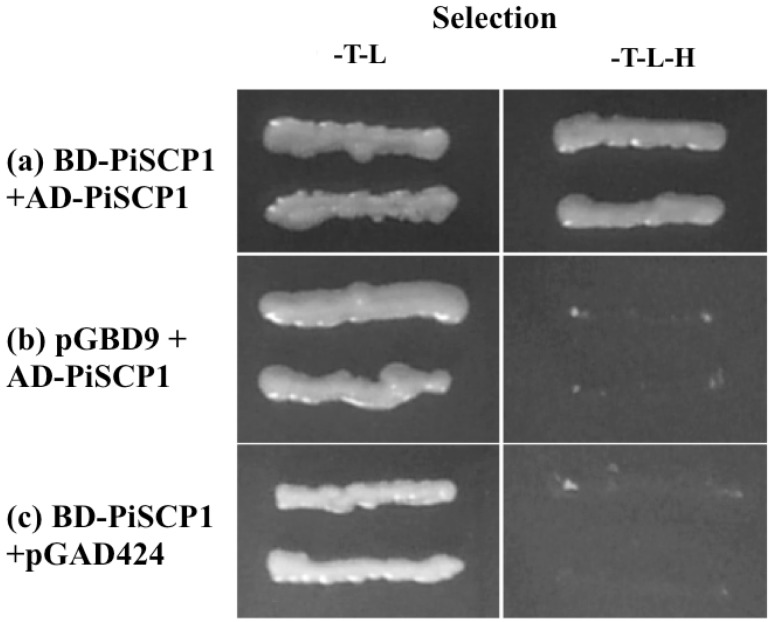
PiSCP1 interacts with itself in the yeast two hybrid assay. Yeast transformed with the bait and prey constructs, was grown on selection media, either SD/-T-L or SD/-T-L-H. A–E yeast containing BD-PiSCP1 and AD-PiSCP1 grows on both media. Neither BD-PiSCP1 nor AD-PiSCP1 alone was able to activate the histidine reporter gene to facilitate growth under histidine selection.

A yeast 2 hybrid screen, using PiSCP1 as bait, yielded a single class of positive clone. The interaction was verified by re-transforming the bait and prey plasmids into yeast and repeating the assay (Figure 6). Sequencing this class determined that it contained PiSCP1 itself. Whilst not providing additional information concerning the biochemical function of PiSCP1 function, this result suggests that PiSCP1 may be capable of forming homodimers or higher-level multimers. That it did this in the absence of either CDPK suggests that this characteristic is not phosphorylation dependent, though we cannot rule out the possibility that a yeast protein kinase might fulfill this activity in the heterologous expression system.

## 3. Experimental Section

### 3.1. Plant Material

*Petunia inflata* was grown under green house conditions with a supplemental light regime of 16 h light and 8 h dark.

### 3.2. Yeast Two Hybrid Library Screening

We used a modified system for yeast two hybrid library screening [42]. The yeast strain AH109 (MATa, trp1-901, leu2-3, 112, ura3-52, his3-200, gal4Δ, gal80Δ, LYS2::GAL1_UAS_-GAL_TATA_-HIS3, MEL1, GAL2_UAS_-GAL2_TATA_-ADE2, URA3::MEL1_UAS_-MEL1_TATA_-lacZ) was used in the screen. AH109 was sequentially transformed first with pGBD/∆N-PiCDPK1 bait construct, and then with a *Petunia inflata* pollen cDNA library in pGAD424 by the lithium acetate method [43]. Transformation was plated onto synthetic drop-out (SD) medium lacking leucine, tryptophan and histidine. After 7 days, cells were replicated onto SD medium lacking leucine, tryptophan, histidine and adenine, and positive transformants were subjected to a colony lift assay as described in yeast protocol handbook (Clontech, Palo Alto., CA, USA). Yeast plasmid DNA was extracted from positive clones and transformed into competent XL1-blue MRF’ (Stratagene, La Jolla, CA, USA) and plated on LB agar plate with 100 µg mL^−1^ ampicillin. Plasmids extracted from XL-blue MRF’ were amplified with pGAD vector primers (GAD-F, 5'-TACCACTACAATGGATGATC-3', GAD-R, 5'-GCACAGTTGAAGTGAACTTG-3') to verify the insert size and amplified fragments were digested with 4-base cutting restriction enzyme, *Taq* I, to group positive clones in classes based on resulting digestion patterns. Positive clones were back-transformed into the AH109 yeast strain with the bait construct to confirm the interaction.

### 3.3. Isolation of Full-Length PiSCP1 cDNA

A *Petunia inflata* flower cDNA library was constructed in λZapII (Stratagene) as previously described [44]. This library was screened to isolate the full-length PiSCP1 cDNA. A 500 bp partial cDNA of PiSCP1 obtained by yeast two hybrid screening was used to synthesize radio-labeled probes using the RTS RadPrime DNA labeling (Life Technologies, Gaithersburg, ML, USA) and hybridized to filters for 16 h in hybridization buffer at 62 °C. After hybridization, filters were washed sequentially with 2× SSC buffer, 0.1% (w/v) SDS; 0.5% (w/v) SDS and 0.1% SSC buffer, 0.1% (w/v) SDS for 20 min per wash. Positive plaque were excised and subjected to secondary screening. Hybridization plaques were purified and *in vivo* excision was performed following the manufacture protocols and the full-length PiSCP1 cDNA was sub-cloned in pGAD vector (Clontech).

### 3.4. RNA Gel Blot Analysis

Total RNA was extracted from 100 mg of *P. inflata* pollen. 15 µg of total RNA was separated on a formaldehyde gel and stained with ethidium bromide to verify equal loading. The RNA was then transferred to Biodyne B membrane (Life Technologies) for hybridization. DNA probes were prepared using the RTS RadPrime DNA labeling (Life Technologies) and hybridized to filters for 16 h in hybridization buffer at 62 °C. After hybridization, filters were washed sequentially with 2× SSC buffer, 0.1% (w/v) SDS; 0.5% (w/v) SDS and 0.1% SSC buffer, 0.1% (w/v) SDS for 20 min per wash. Autoradiography was carried out at −70 °C with an intensifying screen. Autoradiographs were scanned using a LIDE flat bed scanner (Canon USA Ins., New York, NY, USA).

### 3.5. Plasmid Constructs for Recombinant Proteins

Standard molecular cloning methods were used and all constructs were confirmed by DNA sequencing. pRSET-B vector (Invitrogen, Carlsbad, CA, USA) was used to express full-length PiCDPK1 and PiSCP1 fusion constructs possessing an *N*-terminal 6× His tag. The coding regions of PiCDPK1 and PiSCP1 were amplified by PCR using the following primers: PiCDPK1-NcoI-5', 5'-CCATGGGGAACTGTTGTTCAAG-3'; PiCDPK1-NcoI-3', 5-CCATGGCAACAAATGACTCCCTCC-3'; PiSCP1-NcoI-5', 5'-CCATGGAGATGAGTTTAAGTTGCTTG-3'; PiSCP1-NcoI-3', 5-CCATGGCTAAGATGAGTTTAGTTGCTTG-3'. Amplified DNA fragments were cloned into pGEM-T Easy vector (Promega, Madison, WI, USA), digested with *Nco* I and sub-cloned into the *Nco* I site of pRSET-B. 

To create constructs for transient expression in pollen, PiCDPK1 and PiSCP1 coding sequences were amplified by PCR. For the Lat52-PiCDPK1-GFP construct PiCDPK1-NcoI-5' and PiCDPK1-NcoI-3' were used. For the Lat52-PiSCP1-GFP construct, PiSCP1-NcoI-5' and PiSCP1-NcoI-3' were used. Amplified DNA fragments were cloned into pGEM-T Easy vector (Promega), digested with *Nco* I and sub-cloned into pBluescript (KS) vector (Stratagene). For co-localization experiments, Lat52-CFP versions of each construct were generated by amplifying CFP fragment from eCFP (Clontech) using CFP-N-NCO 5'-CCATGGTGAGCAAGGGC-3' and CFP-C-SAC 5'-GAGCTCTTATGTCAGTTGGTCATG-3' and cloned into the Lat52 expression vector to replace the coding region of GFP. The PTS2-CFP fusion protein was generated by amplifying the 5' 174 base pairs of *Arabidopsis* *ped1* thiolase that encode the *N*-terminal PTS2 signal sequence [28] using primers Perox-F 5'-ACCATGGAGAAAGCGATCGA-3' and Perox-R 5'-TCCATGGATAGTGGAGTCCTATG-3' and cloned into the *Nco* I site of Lat52-CFP vector.

### 3.6. Expression and Purification of PiCDPK1 and PiSCP1

*E. coli* cells (BL21) transformed with either pRSET-PiCDPK1 or pREST-PiSCP1 were grown overnight at 37 °C in 2 mL of LB/ampicillin (100 µg /mL), transferred to 250 mL of the same media and cultured until the OD_600_ was 0.7. Expression was induced by the addition of 1 mM isopropyl-thio-β-D-galactoside for 2 h at 30 °C. Cells were harvested, resuspended in ice-cold lysis buffer (10 mM imidazole, 0.3 M NaCl, 50 mM phosphate buffer, pH 8.0, 10% glycerol, 100 μg/mL lysozyme, 1 mM PMSF) and sonicated. Fusion proteins were purified by affinity chromatography with nickel resin (Sigma). After loading the column with the recombinant proteins, it was washed with wash buffer (20 mM imidazole, 0.5 mM NaCl, 50 mM phosphate buffer, pH 6.0, 10% glycerol, 1 mM PMSF) and eluted with elution buffer. Protein concentration was determined using Bio-Rad Protein Assay Kit (Bio-Rad, Hercules, CA, USA). Purity and integrity of the recombinant proteins was assessed by SDS-PAGE.

### 3.7. *In Vitro* Biding Assay and *in Vitro* Phosphorylation Assay

The binding assay was performed by mixing 2 µg of His-tag-PiCDPK1 fusion protein attached to phenyl sepharose (Sigma) beads with 2 µg of His-tag-PiSCP1 fusion protein in the presence of 500 µL of binding buffer (20 mM HEPES, pH 7.5, 5 mM MgCl_2_, 1 mM DTT and 0.1% Triton X-100) with 1 mM Ca^2+^. Samples were rotated at 20 rpm for 2 h at 4 °C, pelleted and washed three times with washing butter (20 mM HEPES, pH 7.5, 5 mM MgCl_2_, 0.3 M NaCl, 1 mM DTT and 0.1% Triton X-100). The proteins were eluted in 1× SDS sample buffer and resolved by SDS-PAGE and transferred to polyvinylidene difluoride (PVDF) membrane (Millipore, Bedford, MA, USA). The pull-down samples of 6× His-tag-PiCDPK1 and 6× His-tag-PiSCP1 fusion proteins were analyzed by Western blotting using monoclonal Anti-polyHISTIDINE Clone HIS-1 antibody (Sigma) and anti-mouse IgG alkaline phosphatase conjugated secondary antibody (Sigma).

0.6 µg of His-tag-PiCDPK1 fusion protein was incubated with 0.5 µg of His-tag-PiSCP1 fusion protein in phosphorylation buffer (50 mM HEPES, pH 7.0, 1 mM MgCl_2_, 1 mM DTT) with either 50 µM Ca^2+^ or 1 mM EGTA. The reaction was initiated by the addition of 10 µCi of [γ-^32^P] ATP and incubated for 10 min at room temperature. The reaction was terminated by the addition of 5× SDS sample buffer and electrophoresed on a 10% SDS-PAGE. The gel was blotted to PVDF membrane and exposed to X-ray film.

### 3.8. Transient Expression in Pollen

Transient expression of GFP fusion constructs in pollen was performed as previously described [15].Briefly, *Petunia inflata* pollen was collected from freshly dehisced anthers (10 flowers/bombardment), and suspended by gentle vortexing in 200 μL of pollen germination medium (PGM) (0.01% H_3_BO_3_, 0.02% MgSO_4_, 0.07% CaCl_2_, 15% PEG-4000, 2% sucrose). The pollen was spotted onto a 2.5 cm sq. piece of positively charge nylon membrane in a 9 cm Petri dish.

Microprojectile bombardment was performed using a PDS-1000/He biolistic system (Bio-Rad). Gold particles (1.1 μm diameter) were prepared according to the manufacturer’s protocol using 2 μg of plasmid DNA/0.5 mg of particles. Co-bombardment was achieved by coating particle with 2 μg of each plasmid construct. 0.5 to 1 μg of plasmid DNA were used for co-localization. Bombardments were performed using 1,100 psi rupture disks, a 0.25 inch gap distance and 1 inch particle travel distance. After bombardment, the pollen was washed from the nylon membrane into a Petridish with 4 mL PGM and cultured on an orbital shaker at 100 rpm for 4 h at 30 °C.

Pollen tubes were stained with FM 4-64 according to Parton *et al.* [45]. FM 4-64 (Molecular Probes, Eugene, OR, USA) was added into PGM to a final concentration of 10 μM and visualized after 15 min of gentle shaking.

### 3.9. Analysis of Transformed Pollen Tubes

Epifluorescence microscopy for GFP observation was performed using an Orthomat epifluorescence microscope (Leitz) with a 40×, dry, 0.7 numerical aperture, GFP fluorescence was visualized using a Fluor objective, 480 nm excitation, 500 nm dichroic mirror and >530 nm emission. Images were captured using a Sensys cooled CCD camera (Photometrics, Tucson, AZ, USA).

Confocal images were obtained using either Bio-Rad MRC 600 or Zeiss 510 laser scanning confocal microscope (Carl Zeiss, Thornwood, NY, USA) 488 nm excitation and 515–565 nm emission was used for GFP signal, 543 nm excitation and 560 nm long-pass emission for FM 4-64, 514 nm excitation and 530–560 nm band-pass emission filter for YFP and 458 nm excitation and 450–490 nm band-pass emission filter for CFP. Confocal images were analyzed using the Metamorph v4.5 (Molecular Devices Corp., Downlington, PA, USA) and processed using Adobe Photoshop v5.5 (Adobe Systems Inc., San Jose, CA, USA).

## 4. Conclusions

The data presented in this manuscript provides evidence that a previously unknown pollen specific protein, PiSCP1, interacts with calcium dependent proteins kinases and localizes to peroxisomes. The determination that PiCDPK2 also localizes to peroxisomes suggests that this is the isoform that most likely interacts with PiSCP1 *in vivo* and that these genes are involved in a calcium regulated pathway that regulates peroxisome function. Over-expression of both PiCDPK2 and PiSCP1 are inhibitory to pollen tube growth, but the processes they affect remain a mystery. The observation that over-expression of PiCPDK2 leads to peroxisomes entering pollen tubes tips is intriguing as it has been reported that peroxisomes are usually excluded from the tip region and that an absence of NO (produced by the peroxisomes) is essential to growth [31], however these pollen tubes still grow albeit at a reduced rate and also exhibit a “normal” tip-focused calcium gradient [15]. Hence, additional studies are needed to determine the role of this pathway in peroxisome function(s) and pollen tube growth. 
